# Paradoxical Response During Para-hisian Pacing: What is the Mechanism?

**Published:** 2011-02-08

**Authors:** Anandaraja Subramanian

**Affiliations:** Department of Cardiology, Indira Gandhi Government General Hospital and Postgraduate Institute, Pondicherry, India

**Keywords:** para-hisian pacing, paradoxical response

## Case Presentation

**Figure 1:**
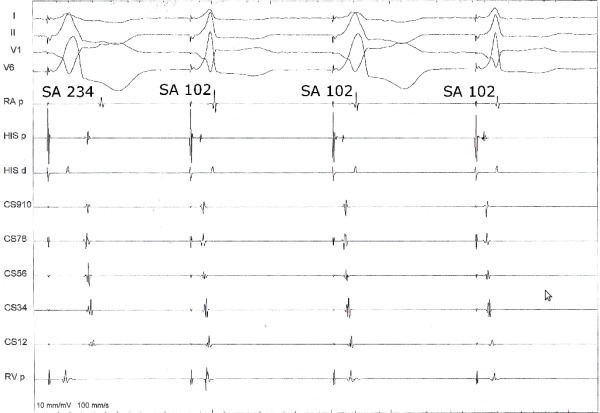
Paradoxical para-Hisian pacing response. In the first two beats, the stimulus to  atrial activation (SA) interval is different between the narrow(His bundle capture) and wide QRS beats (His bundle noncapture), suggesting a nodal response. In the next two beats the stimulus to atrial activation interval is same suggesting an extranodal response. Shown are surface ECG leads I, II, V1 and V6; high atrial electrogram (HRp); His bundle electrograms proximal (His p) and distal (His d); coronary sinus electrograms proximal (CS 9,10) to distal (CS 1,2); and right ventricular apex electrogram (RVp). The His distal recording  shows pacing stimulus and a saturation artifact masking all electrograms due to high current of pacing.

**Figure 2:**
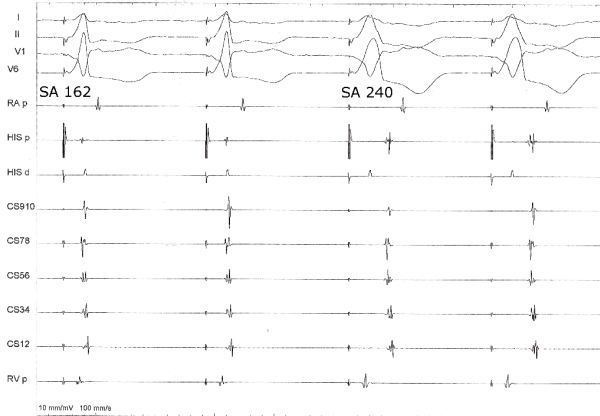
True para-Hisian pacing response. The SA interval during His bundle capture (narrow QRS beats) is longer than the SA interval during His bundle capture in figure 1. Annotations are as in Figure 1.

A 64 year old lady underwent electrophysiologic study for recurrent episodes of palpitation and documented narrow complex tachycardia. 12 lead ECG showed normal sinus rhythm and no evidence of preexcitation. At baseline, the cycle length, AH and HV intervals were 776, 52 and 46 ms respectively. Para-Hisian pacing was done at baseline and the response is shown in Figure 1. The pacing output during the maneuver  was 15 mA. There is evidence of combined His bundle and ventricular capture during the second and fourth complex (narrow complex) and ventricular capture (His bundle non-capture) during the first and third beats (wide complex). There is evidence of both nodal and extranodal response during the maneuver. The first two complexes are suggestive of nodal response and the last 2 complexes are suggestive of extranodal response. On incremental ventricular pacing, VA Wenckeback cycle length was at 340 ms. On programmed ventricular pacing from the right ventricular apex with a single extrastimulus, VA was concentric and decremental. On programmed atrial stimulus, there was AH jump and a narrow complex tachycardia was induced. The septal VA interval during the tachycardia was 30 ms. Entrainment of the tachycardia from the right ventricle resulted in V-A-V response and the PPI-TCL and SAVA intervals were suggestive of typical AVNRT (220 and 154 ms respectively). His refractory PVC failed to advance the atrial activation. Since there was no other evidence of pathway apart from the para-Hisian maneuver, we repeated it and the response is shown in Figure 2. What is the mechanism of the mixed response during the para-Hisian pacing observed earlier?

## Discussion

Para-Hisian pacing is a useful maneuver to distinguish retrograde conduction over the septal accessory pathway and retrograde conduction over the AV node [1]. Pacing is performed near the His bundle or proximal right bundle branch. As the position of the pacing catheter changes subtly during respiration, the pacing stimulus capture shifts between His bundle capture and His-bundle noncapture. The same can be achieved by adjusting the pacing output also. Higher outputs result in His-bundle capture and lower outputs in noncapture. Capture of the His bundle results in relatively narrow QRS complex on the surface ECG, and noncapture results in wide QRS. Comparison of stimulus to atrial (SA) activation times between His-bundle capture and His-bundle noncapture beats can identify the retrograde atrial activation pathway. In the presence of a septal accessory pathway, conduction times are constant during His-bundle capture and His-bundle noncapture. In our patient, initially as seen in figure 1, there is both nodal and extranodal response. The explanation for this could be a presence of a septal pathway producing the extranodal response. The nodal response means that during that time, the atrioventricular nodal conduction has accelerated or the pathway is poorly conducting (and so the conduction occurs through the AV node) producing the nodal response.  The other explanation for nodal response in the presence of a pathway is that the pathway is located at a distance from the pacing site. Since the atrial activation sequence is suggestive of a septal pathway, that possibility is excluded. However subsequently there was no evidence of a pathway during the study as presented earlier. Repetition of the maneuver did not reproduce the extranodal response. Observation of the SA intervals show that there is difference in SA intervals during the His bundle capture (narrow beats) in Figure 1 and Figure 2. The intervals are more in Figure 2. Also the pacing stimulus to atrial electrogram interval was very short in the His bundle proximal recording. This is suggestive of direct atrial capture in Figure 1 (beats 2,3 and 4). Thus the initial response during para-Hisian pacing was misleading. Ablation of the slow pathway was done. Post ablation, no tachycardia could be induced and VA was decremental.

Para-Hisian pacing is a useful maneuver to differentiate retrograde conduction through a pathway versus the AV node. However one has to be careful in performing and interpreting the results. Presence of VA conduction is prerequisite for performing this maneuver and has to be established before performing the maneuver. Otherwise there will be VA dissociation during the maneuver, which can lead to erroneous interpretation especially in the presence of isorhythmic VA dissociation. Also one has to differentiate between a pure Hisian capture and combined His-ventricle capture. Failure to identify will result in misinterpretation of the results [2]. In the present case, there was direct atrial capture which has led to the wrong interpretation. The stimulus to atrial interval in the proximal His bundle recordings during combined His-ventricular capture was 40 and 84 ms during direct atrial capture and retrograde conduction. The early atrial electrogram in the recording from the pacing lead suggests direct atrial capture. Direct atrial capture is quite common during this maneuver. Methods to avoid this include making sure that the pacing lead electrogram has only a small atrial electrogram and use the minimum current possible for His bundle capture. Also the catheter has to be stable during the maneuver, otherwise movement of the catheter can result in direct atrial capture, if the catheter moves into the atrium either with systole or respiration.
